# Data-independent acquisition-based SWATH-MS proteomics profiling to decipher the impact of farming system and chicken strain and discovery of biomarkers of authenticity in organic *versus* antibiotic-free chicken meat

**DOI:** 10.1016/j.crfs.2024.100757

**Published:** 2024-05-01

**Authors:** Laura Alessandroni, Gianni Sagratini, Susana B. Bravo, Mohammed Gagaoua

**Affiliations:** a School of Pharmacy, Chemistry Interdisciplinary Project (CHIP), University of Camerino, Via Madonna delle Carceri, 62032, Camerino, Italy; b Proteomic Unit, Health Research Institute of Santiago de Compostela (IDIS), Santiago de Compostela, A Coruña, Spain; c PEGASE, INRAE, Institut Agro, 35590, Saint-Gilles, France

**Keywords:** Poultry muscle proteome, Farming systems, Gel-based proteomics, SWATH-MS, Omics, Organic meat, Biological pathways

## Abstract

In the literature, there is a paucity of methods and tools that allow the identification of biomarkers of authenticity to discriminate organic and non-organic chicken meat products. Shotgun proteomics is a powerful tool that allows the investigation of the entire proteome of a muscle and/or meat sample. In this study, a shotgun proteomics approach using Sequential Window Acquisition of All Theoretical Mass Spectra (SWATH-MS) has been applied for the first time to characterize and identify candidate protein biomarkers of authenticity in post-mortem chicken *Pectoralis major* muscles produced under organic and non-organic farming systems (antibiotic-free). The proteomics characterization was further performed within two chicken strains, these being Ross 308 and Ranger Classic, which differ in their growth rate. From the candidate protein biomarkers, the bioinformatics enrichment analyses revealed significant differences in the muscle proteome between the two chicken strains, which may be related to their genetic background and rearing conditions. The results further provided novel insights on the potential interconnected pathways at interplay that are associated with the differences as a consequence of farming system of chicken strain, such as muscle contraction and energy metabolism. This study could pave the way to more in-depth investigations in proteomics applications to assess chicken meat authenticity and better understand the impact of farming systems on the chicken muscle and meat quality.

## Highlights

• First application of SWATH-MS proteomics in chicken meat research.
• Towards a proteomics approach for organic chicken meat characterization and authentication.
• First shotgun proteomics study comparing chicken strains and farming systems.
• Discovery of molecular signatures related to chicken strain and farming system.
• Organic farming system can impact chicken proteome more than non-organic one.

## Graphical abstract

**Figure:**
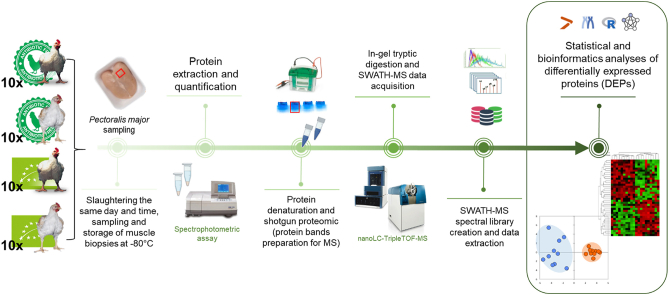

## 1 Introduction

Nowadays, consumers have concerns about both the quality of animal (food) products and their sustainability and they are willing to pay more for guaranteed and certified quality of animal products (Kaygisiz et al., 2019; Nguyen et al., 2019). The exponential increase in the market for organic products in recent years is in line with the emerging expectation of consumers of better quality of animal products (Roy et al., 2023). In Europe, organic meat production is regulated by Regulation (EC) no. 834/2007 (2007) and two implementing regulations, no. 889/2008 and n. 1235/2008. These guidelines regulate the meat supply chain, from origin and breeding of animals, feed, veterinary treatments, slaughtering methods, packaging solutions, transport, storage, import and export of products themselves and their derivatives (European Commission, 1235). Among the variety of meat-based foods on the market, poultry meat is the primary consumed meat, hence constituting a major protein source for people in the most areas of the world (Zaboli et al., 2019; Alessandroni et al., 2022; Feknous et al., 2023).

Modern chicken breeds are the result of decades of artificial selection for commercial purposes (Tallentire et al., 2016). In fact, the most widely used strain in broiler production is the Ross 308 fast growing hybrid, raised to produce high amount of lean muscle (meat) in a short period of rearing time (Karlsson 2016). Inversely, organic production involves longer rearing periods, which causes several welfare problems for fast-growing broilers, so the choice of more suitable strains for this production system is an open issue among poultry industries (Sirri et al., 2011; Rocchi et al., 2021). Ranger Classic slower growth-rate meets more the rearing times required by organic farming, without any feed restrictions than the fast growing strains, which are more adapted to conventional farming systems (Karlsson 2016; Aviagen Group, 2018). Moreover, it has been well documented that pre-slaughter stressors, such as farming conditions, temperature and handling procedures, affect physiological and metabolic functions of animals, with consequent repercussions on both the muscle properties and meat quality (Schwartzkopf-Genswein et al., 2012; Xing et al. 2019, 2020; Terlouw et al., 2021; Alessandroni et al., 2023).

Over the past two decades, foodomics, especially proteomics, have gained huge interest and application in meat research for the study of both quality traits and meat authenticity (Gagaoua and Picard 2022; Gagaoua et al., 2024). Such approaches have been used for several objectives, but mainly to investigate the underlying biological mechanisms involved in meat quality traits determination (della Malva et al., 2022a; della Malva et al., 2022b; Gagaoua et al., 2023). Moreover, proteomics is a very effective tool to analyze the dynamic biochemical changes in post-mortem muscle including the interactions among the proteins (Lamri et al., 2023a). Techniques based on the combination of proteomics with mass spectrometry (MS) can provide a superior ability to separate and identify a large number of muscle proteins with a greater resolving power. Therefore, the application of these techniques can allow a deeper knowledge of the muscle-to-meat conversion mechanisms and their influence on meat quality traits (Picard and Gagaoua, 2020).

Despite that poultry meat is one of the most consumed meats in the world, to date there has been a paucity of published literature on the application of high-throughput omics methods, such as proteomics, to study the impact of farming system on the muscle and/or poultry meat. Thus, in-depth understanding of the protein dynamics related to different growth-rates, diet, strains and farming systems could lead to characterize the potential impacts of these parameters on the muscle proteome and ultimately on chicken meat quality. Therefore, the starting hypotheses of this study were the possibility to investigate the proteome of chicken meat from different strains and farming systems and to discover protein biomarkers of authenticity. To achieve this aim, firstly, a traditional proteomic approach based on two-dimensional electrophoresis combine with MS was applied (Alessandroni et al., 2024). In the present study, we applied for the first time a more powerful approach in the frame of shotgun proteomics, using Sequential Window Acquisition of All Theoretical Mass Spectra (SWATH-MS), to expand on the gained knowledge and to better understand the impacts of farming systems (organic *versus* antibiotic-free farming system) and chicken strains (Ross 308 *versus* Ranger Classic) on the early post-mortem *Pectoralis major* muscle proteomes. Moreover, the identification of candidate protein biomarkers of authenticity of both the farming systems and chicken strains may have important implications for poultry industry in helping producers to ensure consistent high quality of their products. Furthermore, involving biomarkers to authenticate chicken meat products can help in building consumer confidence, while regulators and law enforcement officials can detect and prevent food fraud.

## 2 Materials and methods

### 2.1 Animals and muscle tissue sampling

**Fig. 1:**
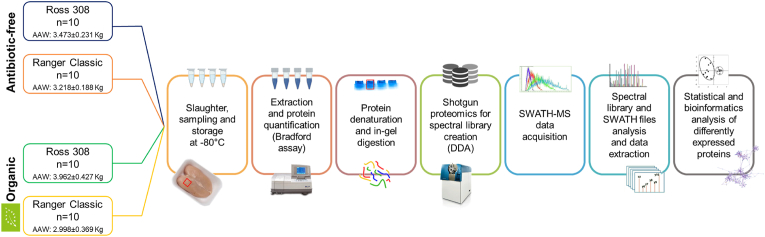
Workflow of the bottom-up shotgun proteomics method applied for the profiling of the 40 chicken *Pectoralis major* samples. (AAW: Average Alive Weight).

Forty chickens, half of which were Ross 308 and half Ranger Classic, were used in this trial for shotgun proteomics analysis. The animals reared under comparable conditions within each condition and slaughtered within the same batch and day were selected and provided by Fileni® industry (Cingoli, Italy). From each strain, 10 chickens were reared under an antibiotic-free inside ground farming and 10 others according to European standards of organic farming and livestock (European Commission, 1235). Fig. 1 shows the 4 groups of samples, divided according to strains and farming system: **ORO:** Organic Ross 308, **ORA:** Organic Ranger Classic, **ARO**: Antibiotic-free Ross 308 and **ARA:** Antibiotic-free Ranger Classic. *Pectoralis major* muscle biopsies were collected immediately after slaughter at a similar sampling time of 3h to avoid any variation. A 2 cm^3^ section was cut using a sterile scalpel from the top right part of each breast with randomization, frozen in liquid nitrogen and stored at −80 °C until protein extraction. The workflow depicting the different steps followed in this work to apply a shotgun proteomics using a SWATH-MS approach is given in Fig. 1.

### 2.2 Extraction of chicken muscle proteins and quantification

For the total protein extraction, 200 mg of muscle tissue were homogenized using a T 25 digital Ultra-Turrax® in 3 mL of fresh extraction buffer containing 8.3 M urea, 2 M thiourea, 1% Dithiothreitol (DTT), 2% CHAPS (3-[(3-cholamidopropyl) dimethylammonio]-1-propanesulfonate) and 2% Pharmalyte® (Immobilized pH gradient (IPG) buffer pH 3–10) (Bouley et al., 2004; Lamri et al., 2023b). Protein homogenates were then incubated for 30 min on wet-ice and centrifuged for 30 min at 10000 rpm at 4 °C (Gagaoua et al., 2021b). Subsequently, the supernatants were transferred into Eppendorf tubes and stored at −80 °C until protein quantification and further separation analysis.

The quantification of the total protein concentrations of the frozen protein extracts were determined using the Bradford dye-binding method (Bradford 1976). For this purpose, the Biorad protein assay kit (Bio-Rad Laboratories, Hercules, CA, USA) has been used and serum albumin from bovine (BSA) was used as a standard at a concentration of 1 mg/mL the measurement was performed using an UV-1700 spectrophotometer (Pharmaspec, Shimadzu).

### 2.3 Protein bands preparation by SDS-PAGE electrophoresis for LC-MS/MS

Before the separation of the protein extracts using one-dimensional (1D) electrophoresis, the muscle protein extracts were first denatured by 1:1 Laemmli sample buffer (2 × concentrate, #S3401, Sigma-Aldrich, Saint Louis, USA) and diluted to equivalent protein content. The Laemmli buffer contains 4% w/v SDS, 125 mM Tris (pH 6.8), 20% v/v glycerol, 10% v/v β-mercaptoethanol and 0.004% bromophenol blue. The samples were vortexed and incubated at room temperature for 5 min before heating at 95 °C for 15 min using a standard block heater (VWR, International). The denatured proteins were then assessed by SDS-PAGE gel electrophoresis on 12% acrylamide gels to check the protein quality before running the shotgun proteomics protocol.

The denatured protein extracts (30 μg, final volume of 15 μL) were loaded in each lane of standard 12% resolving and 4% stacking gels of one-dimensional SDS-PAGE (sodium dodecyl sulphate polyacrylamide gel electrophoresis) using a Mini-PROTEAN Tetra Cell system (Bio-Rad Laboratories, Hercules, CA, USA) at 5 W during about 15 min to stack and concentrate the proteins in the stacking gel. The 40 samples were all run under the same electrophoresis −4 freshly prepared gels). After the concentration step, the gels were subsequently washed with MilliQ water, stained with EZ Blue Gel staining reagent (Sigma-Aldrich, St. Louis, USA) and kept in gentle agitation for 2 h and then washed with MilliQ water. The visible protein bands were excised with a sterile disposable scalpel and transferred into Eppendorf tubes that contains 150 μL of 50 mM ammonium bicarbonate and 50% ethanol. Then, disulfide bonds were reduced with 200 μL of 10 mM dithiothreitol (Sigma-Aldrich, St. Louis, USA) in 50 mM ammonium bicarbonate buffer for 45 min at 56 °C. Proteins alkylation was carried out with 200 μL of 55 mM iodoacetamide (Sigma-Aldrich, St. Louis, USA) in 50 mM ammonium bicarbonate buffer for 30 min in darkness. Subsequently, the protein bands were destained by 200 μL of 25 mM ammonium bicarbonate (Sigma-Aldrich, St. Louis, USA), 5% acetonitrile (ACN) for 30 min and washed twice with 200 μL of 25 mM ammonium bicarbonate, 50% ACN for 30 min each time. Finally, bands were dehydrated with 100% ACN for 10 min then the liquid was discarded. The dried protein bands were stored at −80 °C until SWATH-MS analysis.

### 2.4 LC-MS/MS analysis and protein identification and quantification by SWATH-MS

In order to make global protein identification and quantification using SWATH-MS approach, an equal amount of protein (40 μg) from pooled samples of each group (ORO, ORA, ARO and ARA) were loaded on a 10% SDS-PAGE gel. The run was stopped as soon as the front had penetrated 3 mm into the resolving gel (Bonzon-Kulichenko et al., 2011). The protein band was detected by Sypro-Ruby fluorescent staining (Lonza, Switzerland), excised, and processed for in-gel, manual tryptic digestion as described elsewhere (Shevchenko et al., 1996). Peptides were extracted by carrying out three 20 min incubations in 40 μL of 60% acetonitrile dissolved in 0.5% HCOOH. The resulting peptide extracts were pooled, concentrated in a SpeedVac, and stored at −20 °C.

### 2.5 Protein quantification by SWATH-MS (Sequential Window Acquisition of all Theoretical Mass Spectra)

#### 2.5.1 Creation of a spectral library

To construct the MS/MS spectral libraries, the peptide solutions were analyzed by a data-dependent acquisition (DDA) approach using a micro-LC-MS/MS. To get a good representation of the peptides and proteins present in all the samples, pooled vials of samples from each group (ORO, ORA, ARO and ARA) were prepared using equal mixtures of the original samples. Therefore, 4 μL (4 μg) of each pool was separated into a micro-LC system Ekspert nLC425 (Eksigen, Dublin, CA, USA) using a column Chrom XP C18 150 × 0.30 mm, 3 μm particle size and 120 Å pore size (Eksigent, SCIEX) at a flow rate of 5 μL/min. Water and ACN, both containing 0.1% formic acid, were used as solvents A and B, respectively. The gradient run consisted of 5%–95% B for 30 min, 90% B for 5 min and finally 5 min at 5% B for column equilibration, for a total run time of 40 min. When the peptides eluted, they were directly injected into a hybrid quadrupole-TOF mass spectrometer Triple TOF 6600plus (Sciex, Redwood City, CA, USA) operated with a data-dependent acquisition (DDA) system in positive ion mode. A Micro source (Sciex) was used for the interface between microLC and MS, with an application of 2600 V voltage. The acquisition mode consisted of a 250 ms survey MS scan from 400 to 1250 m/z followed by an MS/MS scan from 100 to 1500 m/z (25 ms acquisition time) of the top 65 precursor ions from the survey scan, for a total cycle time of 2.8 s. The fragmented precursors were then added to a dynamic exclusion list for 15 s; any singly charged ions were excluded from the MS/MS analysis.

The peptide and protein identifications were performed using Protein Pilot software (version 5.0.1, Sciex, Redwood City, CA, USA). Data were searched using a chicken specific Uniprot database, specifying iodoacetamide as Cys alkylation. This software uses the algorithm ParagonTM for database search and ProgroupTM for data grouping. This false discovery rate was performed using a non-linear fitting method displaying only those results that reported a 1% global false discovery rate or better for both peptides and proteins (Shilov et al., 2007). The MS/MS spectra of the identified peptides were then used to generate the spectral library for SWATH peak extraction using the add-in for PeakView Software (version 2.2, Sciex, Redwood City, CA, USA) and MS/MSALL with SWATH Acquisition MicroApp (version 2.0, Sciex, Redwood City, CA, USA). Only peptides with a confidence score above 99% (as obtained from Protein Pilot database search) were included in the spectral library.

#### 2.5.2 Relative quantification by SWATH-MS acquisition

SWATH-MS acquisition was performed on a TripleTOF® 6600plus LC-MS/MS system (AB Sciex, Redwood City, CA, USA). In this case, the samples were analyzed using a data-independent acquisition (DIA) method by considering the 40 samples. Each sample (4 μL) was analyzed using the LC-MS equipment and LC gradient described above for building the spectral library but instead using the SWATH-MS acquisition method. The method consisted of repeating a cycle that consisted of the acquisition of 100 TOF MS/MS scans (400–1500 m/z, high sensitivity mode, 50 ms acquisition time) of overlapping sequential precursor isolation windows of variable width (1 m/z overlap) covering the 400–1250 m/z mass range with a previous TOF MS scan (400–1500 m/z, 50 ms acquisition time) for each cycle. Total cycle time was 6.3 s. For each sample set, the width of the 100 variable windows was optimized according to the ion density found in the DDA runs using a SWATH variable window calculator worksheet from Sciex.

#### 2.5.3 Library data analysis using DIA-NN software

**Fig. 2:**
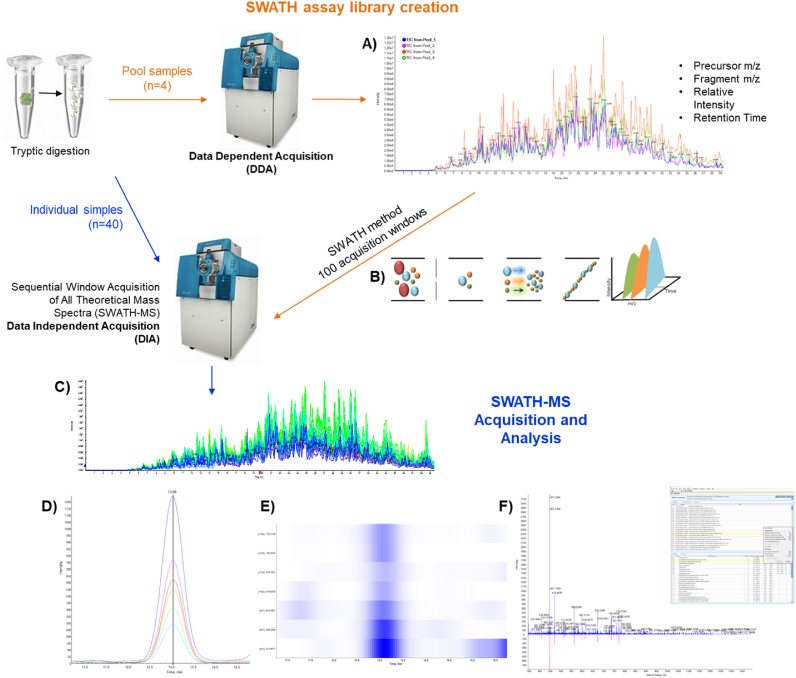
SWATH-MS data-independent acquisition and targeted data analysis. **A)** Overlap of SWATH TIC (Total Ion Chromatogram) obtained from the four pool samples. **B)** Data-independent acquisition (DIA) method are composed of consecutive acquisition of high resolution and accurate mass fragment ion spectra during the 40 min that is an entire chromatographic elution time in our system (retention time). This method is made by repeatedly stepping through 100 discrete precursor isolation in 100 windows of 25-Da width across the 300–1500 m/z range. **C)** Overlap of SWATH TIC profile obtained from all individual samples. **D)** Representation of the peptide ADEGDYTVEATNESGR and its 7 transitions from the IGFN1 protein, as an example. SWATH area extraction consists of retrieving the most intense 10 peptides of the all proteins and their 7 more intense fragment ions from a spectral library. In this case, a peptide is highlighted at 13 min and the peak and the ion fragments displaying co-eluting characteristics. Extraction of the fragment ions of all proteins in the library enables accuracy protein quantitation. **E)** Heatmap showing the coalition of the peptide and the fragments. **F)** MS/MS spectrum from the peptide ADEGDYTVEATNESGR in the SWATH DIA experiment.

After the samples were acquired individually using the SWATH method, DIA-NN (1.8) was used to generate the protein values per samples using recommended settings (Demichev et al., 2020). Mass ranges and charges were set appropriately: peptide length range 7–35, precursor charge range 2–5, precursor range 350–1400 m/z and fragment ion range 100–1500 m/z. For the search, chicken proteome database from Uniprot was added. Other important parameters were: for neural network classifier it was set as single pass mode, for the quantification strategy it was set for any LC (high accuracy) and for the cross-run normalization, it was set as RT-Dependent. For the in silico predicted library search, the reduced memory option was additionally activated. The proteins were quantified with filtering criteria of 10 peptides/protein and 7 transitions of the fragments/peptide at an FDR of 1.0 % (Fig. 2).

The integrated peak areas obtained from DIA-NN were exported to the MarkerView software (Sciex, Redwood City, CA, USA) for relative quantitative analysis. The export generated information about individual ions, the summed intensity of different ions for a particular peptide and the summed intensity of different peptides for a particular protein. MarkerView has been used for analysis of SWATH-MS data reported in other proteomics studies because of its data-independent method of quantitation (Luo et al., 2017; Meyer and Schilling 2017; Ortea et al., 2018; Tan and Chung 2018). MarkerView uses processing algorithms that accurately find chromatographic and spectral peaks directly from the raw SWATH data. Data alignment by MarkerView compensated for minor variations in both mass and retention time values, hence ensuring that identical compounds in different samples were accurately compared to each other. To control for possible uneven sample loss across the different samples during the sample preparation process, we performed an MLR (multivariate linear regression) global normalization. Unsupervised multivariate statistical analysis using principal component analysis (PCA) was performed to compare the data across samples. The average MS peak area of each protein was derived using the MarkerView from the replicates of the SWATH-MS of each sample followed by Student's t-test analysis software for comparison among the samples based on the averaged area sums of all the transitions derived for each protein. The *t*-test indicated how well each variable distinguishes the two groups, reported as a p-value. For each library, we set for the differentially abundant proteins a p-value <0.05 and a 1.5-fold in both increase or decrease direction.

### 2.6 Statistical analysis

The statistical analyses were processed using the XLSTAT 2018.2 software (AddinSoft, Paris, France) and the webservice MetaboAnalyst 5.0 platform (https://www.metaboanalyst.ca/). First, the missing values were estimated using the k-nearest neighbor (KNN) algorithm. The data were subsequently normalized using a log2 transformation and Pareto scaling approach. Then, a one-way ANOVA was used to identify the differentially abundant proteins for each pairwise comparison between the groups (ORO: Organic Ross 308, ORA: Organic Ranger Classic, ARO: Antibiotic-free Ross 308 and ARA: Antibiotic-free Ranger Classic). Protein abundances were considered differently abundant at p < 0.05. Subsequently, all proteins characterized as differentially abundant in the four comparisons were analyzed using principal component analysis (PCA) as previously described (Zhu et al., 2021). The PCA analysis aimed to visualize the distribution of the differentially abundant proteins (candidate protein biomarkers) within the corresponding strains or farming systems. The PCA further aimed to screen the potential of the protein biomarkers to accurately separate the groups (individuals) within the corresponding bi-plots. To check the suitability of the factorial model, the Kaiser-Meyer-Olkin (KMO) test for sampling adequacy was used and the overall KMO values are reported for each PCA. To further analyze and better visualize the individual variability within the groups, statistical heatmaps using hierarchical clustering analysis were generated and visualized on the differentially abundant proteins using Heatmapper online tool (Babicki et al., 2016). Protein expression values were log2-normalized and cluster analysis was performed using Z-score. Average linkage for clustering method and Euclidean distance measurement methods were the conditions applied to produce the double hierarchical dendrograms.

### 2.7 Bioinformatics analysis

The bioinformatics analyses were performed on the protein lists to identify the main molecular and biological functions using Gene Ontology (GO) analyses to highlight the major and related molecular signatures as previously described (Gagaoua et al., 2021a). Metascape®, an open-source tool (https://metascape.org/) was used to functionally categorize and identify the significant and enriched GO terms, and to investigate the pathways and process enrichments using the changing proteins for each condition. The tool combines a hypergeometric test and Benjamini−Hochberg p-value correction algorithm to display the first statistically significant enriched ontology terms. From the same analysis, we also generated the enriched GO networks to decipher the degree of interconnectedness among the pathways. Furthermore, the up and down regulated proteins were compared using GO hierarchical heatmaps to depict the similarities and divergences among the enriched GO terms (Gagaoua et al., 2021c).

## 3 Results

The SWATH-MS proteomics approach applied in this study on 40 individual samples from four experimental groups allowed the quantification of 660 proteins in the chicken *Pectoralis major* muscle samples. The relative abundances of the muscle proteomes were compared based on the different conditions investigated in this trial, these being within the strain under the same farming system or within the farming system under the same chicken strain. The results of the different comparisons are given and presented in the following subsections.

### 3.1 Comparison of the muscle proteome of Ranger Classic and Ross 308 reared under antibiotic-free farming system

**Fig. 3:**
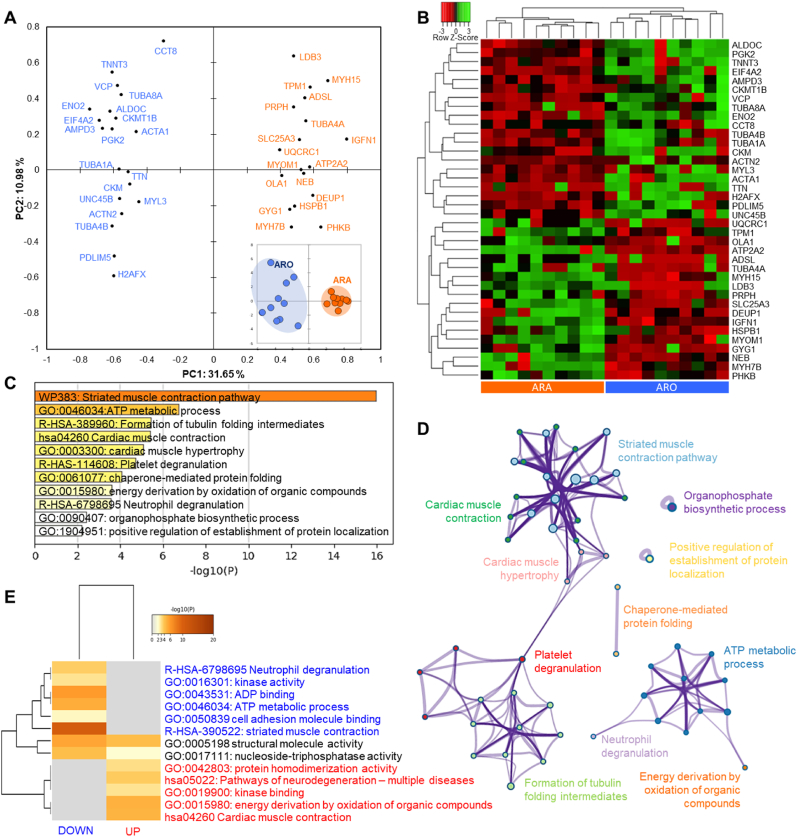
Statistical and bioinformatics analyses on the differentially expressed proteins (DEPs = 38) between Ross 308 and Ranger Classic antibiotic-free chicken *post-mortem* muscle proteome. **A)** Principal Component Analysis (PCA) highlighting the distribution of the 38 DEPs and the separation of the two groups in the bi-plot in the bottom right (KMO score = 0.64). **B)** Heatmap of DEPs analyzed by hierarchical clustering. Each row represents a single protein. Each column represents an individual chicken. Protein expression values were log2-normalized and cluster analysis was performed using Z-score. Red indicates a low expression level; green indicates a high expression level. **C-E)** Bioinformatic enrichment analyses (Gene Ontology, KEGG, Reactome) on the 38 DEPs. **C)** Top enriched terms. **D)** Network layout based on the pathways of the 38 DEPs. Each term is represented by a circle node, where its size is proportional to the number of input genes fall under that term, and its color represent its cluster identity. Terms with a similarity score >0.3 are linked by an edge (the thickness of the edge represents the similarity score). **E)** Hierarchical Heatmap clustering comparing the UP (n = 18) and DOWN (n = 20) DEPs in terms of the significant process and pathways among the top Gene Ontology terms and colored according to P-values: terms with a P-value <0.01, a minimum count of 3, and an enrichment factor >1.5. Colors from grey to brown indicate p-values from high to low; and grey cells indicate the lack of significant enrichment. The terms in blue color are specific to down-regulated proteins, those in red are for up-regulated proteins in Ranger Classic chickens and those in black are significant and common to both protein lists. (For interpretation of the references to color in this figure legend, the reader is referred to the Web version of this article.)

The comparison of Ranger Classic (ARA) and Ross 308 (ARO) chicken strains reared under antibiotic-free farming system revealed 38 differentially expressed proteins (DEPs) from which 18 were up- and 20 down-regulated in ARA (Fig. 3 and Table S1). The projection of the DEPs proteins by means of a principal component analysis (PCA) allowed a clear separation of ARA from ARO (Fig. 3a). This is further evidenced by the hierarchical clustering analysis at the individual level by means of the clustering analysis depicting in the heatmap (Fig. 3b) the individual abundances.

The bioinformatics enrichment analyses on the 38 DEPs through Gene Ontology (GO), KEGG and Reactome databases generated using Metascape® webservice tool are given in Fig. 3c–e. The enrichment analysis revealed 11 cluster terms that are significantly enriched (Fig. 3c) mainly dominated by “striated muscle contraction pathway (WP383)” followed by “ATP metabolic process (GO:0046034)” and “formation of tubulin folding intermediates (R-HSA-389960)” as the top three GO terms. These enriched cluster terms allowed to construct a process network of the pathways and molecular signatures characterizing the differentially expressed proteins (Fig. 3d), which further depicts the degree of interconnectedness among the molecular signatures. In fact, it confirmed the dominance of a sub-network of muscle structure, contraction and associated pathways that interacts with platelet degranulation and formation of tubulin folding intermediates and another one related to ATP metabolic process and energy derivation by oxidation of organic compounds.

The in-depth comparison of the up- and down-regulated proteins in terms of enriched terms is given by means of a heatmap (Fig. 3e). The common and specific biological pathways to the two protein lists are evidenced. Only two enriched terms were found to be common: “structural molecule activity (GO:0005198)” and “nucleoside-triphosphatase activity (GO:0017111)”. Six pathways were specific and down-regulated in Ranger Classic: “neutrophil degranulation (R-HSA-6798695)”, “kinase activity (GO:0016301)”, “ADP binding (GO:0043531)”, “ATP metabolic process (GO:0046034)”, “cell adhesion molecule binding (GO:0050839)” and “striated muscle contraction (R-HSA-390522)”. However, five pathways were specific and up-regulated in Ranger Classic: “protein homodimerization activity (GO:0042803)”, “pathways of neurodegeneration – multiple diseases (hsa05022)”, “kinase binding (GO:0019900)”, “energy derivation by oxidation of organic compounds (GO:0015980)” and “cardiac muscle contraction (hsa04260)”.

### 3.2 Comparison of the muscle proteome of Ranger Classic and Ross 308 reared under organic farming system

**Fig. 4:**
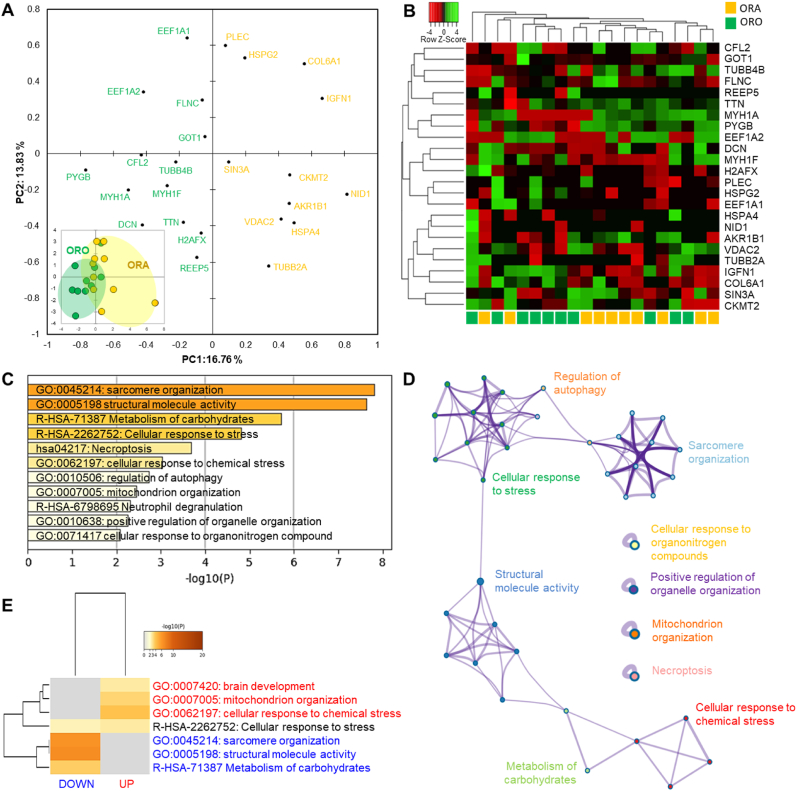
Statistical and bioinformatics analyses on the differentially expressed proteins (DEPs = 24) between Ross 308 and Ranger Classic organic chicken *post-mortem* muscle proteome. **A)** Principal Component Analysis (PCA) highlighting the distribution of the 24 DEPs and the separation of the two groups in the bi-plot in the bottom right (KMO score = 0.25). **B)** Heatmap of DEPs analyzed by hierarchical clustering. Each row represents a single protein. Each column represents an individual chicken. Protein expression values were log2-normalized and cluster analysis was performed using Z-score. Red indicates a low expression level; green indicates a high expression level. **C-E)** Bioinformatic enrichment analyses (Gene Ontology, KEGG, Reactome) on the 24 DEPs. **C)** Top enriched terms. **D)** Network layout based on the pathways of the 24 DEPs. Each term is represented by a circle node, where its size is proportional to the number of input genes fall under that term, and its color represent its cluster identity. Terms with a similarity score >0.3 are linked by an edge (the thickness of the edge represents the similarity score). **E)** Hierarchical Heatmap clustering comparing the UP (n = 13) and DOWN (n = 11) DEPs in terms of the significant process and pathways among the top Gene Ontology terms and colored according to P-values: terms with a P-value <0.01, a minimum count of 3, and an enrichment factor >1.5. Colors from grey to brown indicate p-values from high to low; and grey cells indicate the lack of significant enrichment. The terms in blue color are specific to down-regulated proteins, those in red are for up-regulated proteins in Ranger Classic chickens and those in black are significant and common to both protein lists. (For interpretation of the references to color in this figure legend, the reader is referred to the Web version of this article.)

The comparison of Ranger Classic (ORA) and Ross 308 (ORO) chicken strains reared under organic farming system, revealed 24 differentially expressed proteins (DEPs) from which 13 were up- and 11 down-regulated in ARA (Fig. 4 and Table S2). The number of proteins was less than the previous comparing under antibiotic-free farming system. The projection of the DEPs proteins by means of a PCA did not clearly separate the two groups (Fig. 4a). In fact, few chickens overlap between the ORA and ORO. This weak separation was further evidenced by the hierarchical clustering analysis through the heatmap using the individual abundances of the DEPs (Fig. 4b).

The bioinformatics enrichment analyses on the 24 DEPs through Gene Ontology (GO), KEGG and Reactome databases are given in Fig. 4c. It resulted that 11 cluster terms were significantly enriched mainly dominated by “sarcomere organization (GO:0045214)” and “structural molecule activity (GO:0005198)” followed by “metabolism of carbohydrates (R-HSA-71387)” and “cellular response to stress (R-HSA-2262752)” as the top four GO terms. These enriched cluster terms allowed also to build a process network of the enriched molecular signatures pathways (Fig. 4d). It confirmed the dominance of a sub-network sarcomere organization and structural molecule activity pathways which interacts with another one related to cellular response to stress processes and carbohydrate metabolism.

Compared to the previous comparison, the heatmap of the up- and down-regulated proteins in the case of organic farming revealed less enriched terms (Fig. 4e). Only one enriched term was found to be common being “cellular response to stress (R-HSA-2262752)”, which was found to be likely more significant in the up-regulated protein list. Three pathways were specific and down-regulated in Ranger Classic: “sarcomere organization (GO:0045214)” and “structural molecule activity (GO:0005198) both highly enriched followed by “metabolism of carbohydrates (R-HSA-71387)”. However, three pathways were specific and up-regulated in Ranger Classic these being “brain development (GO:0007420)”, “mitochondrion organization (GO:0007005) and “cellular response to chemical stress (GO:0062197)”.

### 3.3 Comparison of the muscle proteome of Ross 308 reared under organic and antibiotic-free farming systems

**Fig. 5:**
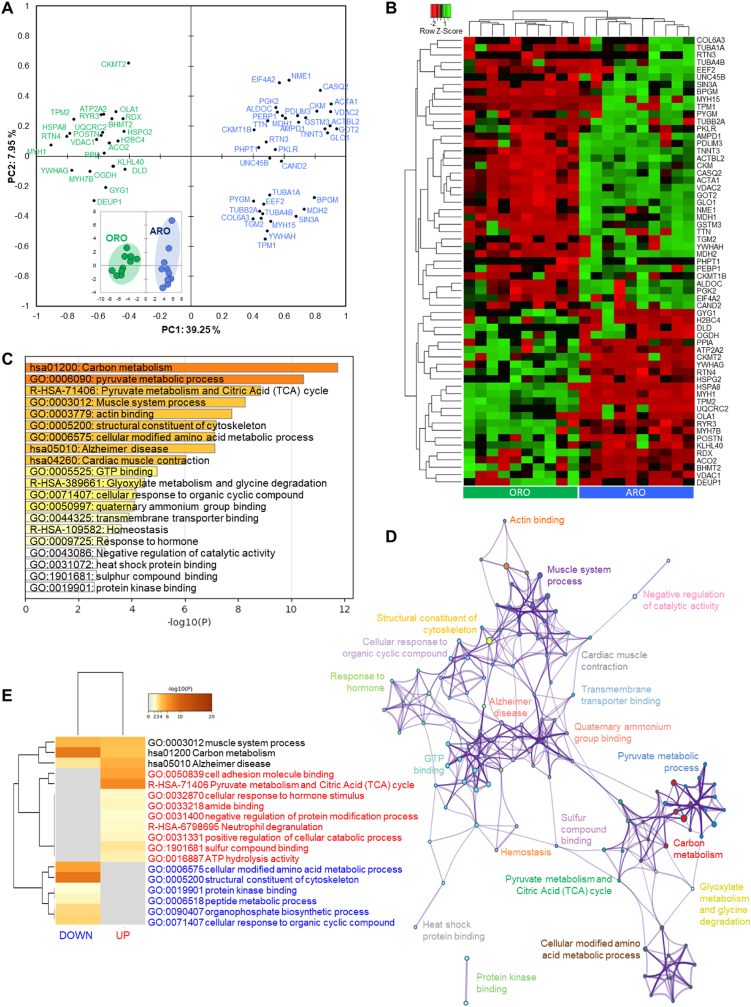
Statistical and bioinformatics analyses on the differentially expressed proteins (DEPs = 61) between organic and antibiotic-free Ross 308 chicken *post-mortem* muscle proteome. **A)** Principal Component Analysis (PCA) highlighting the distribution of the 61 DEPs and the separation of the two groups in the bi-plot in the bottom left (KMO score = 0.70). **B)** Heatmap of DEPs analyzed by hierarchical clustering. Each row represents a single protein. Each column represents an individual chicken. Protein expression values were log2-normalized and cluster analysis was performed using Z-score. Red indicates a low expression level; green indicates a high expression level. **C-E)** Bioinformatic enrichment analyses (Gene Ontology, KEGG, Reactome) on the 61 DEPs. **C)** Top enriched terms. **D)** Network layout based on the pathways of the 61 DEPs. Each term is represented by a circle node, where its size is proportional to the number of input genes fall under that term, and its color represent its cluster identity. Terms with a similarity score >0.3 are linked by an edge (the thickness of the edge represents the similarity score). **E)** Hierarchical Heatmap clustering comparing the UP (n = 24) and DOWN (n = 37) DEPs in terms of the significant process and pathways among the top Gene Ontology terms and colored according to P-values: terms with a P-value <0.01, a minimum count of 3, and an enrichment factor >1.5. Colors from grey to brown indicate p-values from high to low; and grey cells indicate the lack of significant enrichment. The terms in blue color are specific to down-regulated proteins, those in red are for up-regulated proteins in organic system and those in black are significant and common to both protein lists. (For interpretation of the references to color in this figure legend, the reader is referred to the Web version of this article.)

The comparison within Ross 308 chicken strain reared under Antibiotic-free (ARO) or Organic (ORO) farming systems revealed 61 differentially expressed proteins (DEPs) from which 24 were up- and 37 down-regulated in ORO (Fig. 5 and Table S3). The projection of the DEPs proteins by means of a PCA allowed a clear separation of ORO from ARO (Fig. 5a). This is further evidenced by the hierarchical clustering analysis through the heatmap using the individual abundances (Fig. 5b).

The bioinformatics enrichment analyses applied to the 61 DEPs through Gene Ontology (GO), KEGG and Reactome databases are given in Fig. 5c. It resulted that 20 cluster terms were significantly enriched mainly dominated by “carbonic metabolism (hsa01200)” and “pyruvate metabolic process (GO:0006090)” followed by “pyruvate metabolism and Citric Acid (TCA) cycle (R-HSA-71406)”, “Muscle system process (GO:0003012)”, “actin binding (GO:0003779)”, “structural constituent of cytoskeleton (GO:0005200)”, “cellular modified amino acid metabolic process (GO:0006575)”, “Alzheimer disease (hsa05010)” and “Cardiac muscle contraction (hsa04260)” as the top GO terms. These enriched cluster terms allowed to construct a complex and larger process network of the related pathways (Fig. 5d), depicting a strong interconnectedness among the pathways and proteins that are involved. The comparison by means of a heatmap of the up- and down-regulated proteins in terms of enriched GO terms is given in Fig. 5e. The analyses evidenced three enriched terms to be common between the two protein lists: “muscle system process (GO:0003012)”, “carbon metabolism (hsa01200)” and “Alzheimer disease (hsa05010)”. Six pathways were specific and down-regulated in organic samples, first dominated by “structural constituent of cytoskeleton (GO:0005200)” and “cellular modified amino acid metabolic process (GO:0006575)” followed by “protein kinase binding (GO:0019901)”, “peptide metabolic process (GO:0006518)”, “organophosphate biosynthetic process (GO:0090407)” and “cellular response to organic cyclic compound (GO:0071407)”. However, nine pathways were specific and up-regulated in organic meat, dominated by “Pyruvate metabolism and Citric Acid (TCA) cycle (R-HSA-71406)”, and “cell adhesion molecule binding (GO:0050839)”, followed by “cellular response to hormone stimulus (GO:0032870)”, “amide binding (GO:0033218)”, “negative regulation of protein modification process (GO:0031400)”, “Neutrophil degranulation (R-HSA-6798695)”, “positive regulation of cellular catabolic process (GO:0031331)”, “sulfur compound binding (GO:1901681)” and “ATP hydrolysis activity (GO:0016887)”.

### 3.4 Comparison of the muscle proteome of Ranger Classic reared under organic and antibiotic-free farming systems

**Fig. 6:**
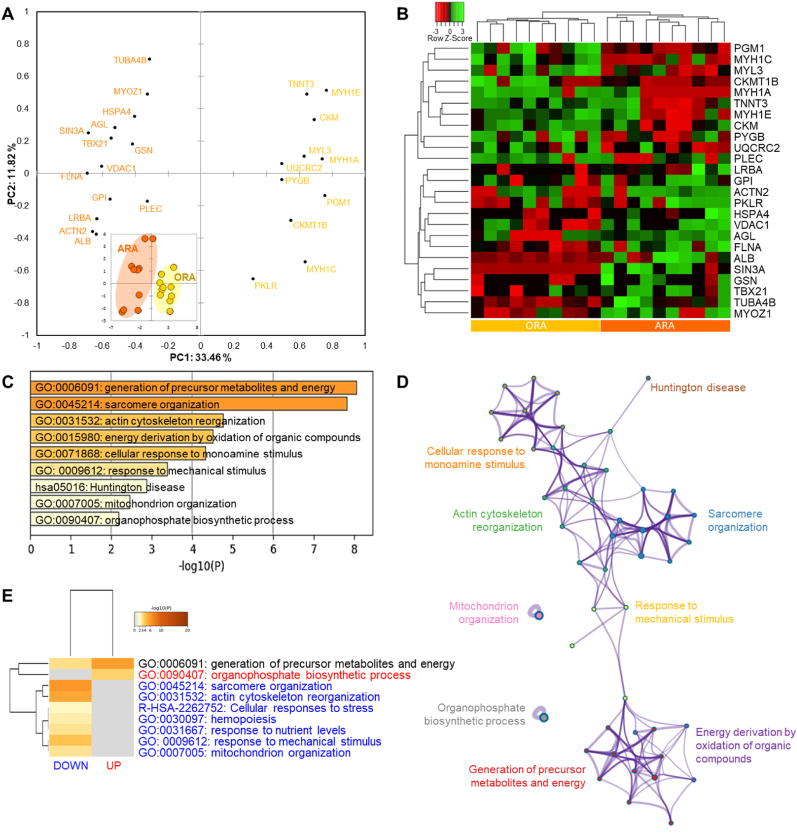
Statistical and bioinformatics analyses on the differentially expressed proteins (DEPs = 25) between organic and antibiotic-free Ranger Classic chicken *post-mortem* muscle proteome. **A)** Principal Component Analysis (PCA) highlighting the distribution of the 25 DEPs and the separation of the two groups in the bi-plot in the bottom left (KMO score = 0.56). **B)** Heatmap of DEPs analyzed by hierarchical clustering. Each row represents a single protein. Each column represents an individual chicken. Protein expression values were log2-normalized and cluster analysis was performed using Z-score. Red indicates a low expression level; green indicates a high expression level. **C-E)** Bioinformatic enrichment analyses (Gene Ontology, KEGG, Reactome) on the 25 DEPs. **C)** Top enriched terms. **D)** Network layout based on the pathways of the 25 DEPs. Each term is represented by a circle node, where its size is proportional to the number of input genes fall under that term, and its color represent its cluster identity. Terms with a similarity score >0.3 are linked by an edge (the thickness of the edge represents the similarity score). **E)** Hierarchical Heatmap clustering comparing the UP (n = 11) and DOWN (n = 14) DEPs in terms of the significant process and pathways among the top Gene Ontology terms and colored according to P-values: terms with a P-value <0.01, a minimum count of 3, and an enrichment factor >1.5. Colors from grey to brown indicate p-values from high to low; and grey cells indicate the lack of significant enrichment. The terms in blue color are specific to down-regulated proteins, those in red are for up-regulated proteins in organic meat and those in black are significant and common to both protein lists. (For interpretation of the references to color in this figure legend, the reader is referred to the Web version of this article.)

The comparison within Ranger Classic chicken strain reared under Antibiotic-free (ARA) or Organic (ORA) farming systems revealed 25 differentially expressed proteins (DEPs, n = 25) from which 11 were up- and 14 down-regulated in ORA (Fig. 6 and Table S4). Although the number of DEPs is smaller, their projection by means of a PCA allowed an acceptable separation of ORA from ARA (Fig. 6a). This is further confirmed by the hierarchical clustering analysis through the heatmap using the individual abundances of the DEPs (Fig. 6b).

The bioinformatics enrichment analyses on the 25 DEPs through Gene Ontology (GO), KEGG and Reactome databases are given in Fig. 6c. The analysis revealed 9 cluster terms to be significantly enriched mainly dominated by “generation of precursor metabolites and energy (GO:0006091)” and “sarcomere organization (GO:0045214)” followed by “actin cytoskeleton reorganization (GO:0031532)” as the top GO terms. These enriched cluster terms allowed to construct a process network connecting seven molecular signatures (Fig. 6D). It confirmed the dominance of a sub-network muscle structure and associated pathways and another one related to energy metabolic processes. Furthermore, the comparison by means of a heatmap of the up- and down-regulated proteins in terms of enriched terms (Fig. 6e), revealed the common and specific biological pathways. One enriched term only was found to be common being “generation of precursor metabolites and energy (GO:0006091)”. A higher number of pathways (n = 7) were specific and down-regulated in organic samples: “sarcomere organization (GO:0045214)”, “actin cytoskeleton reorganization (GO:0031532)”, “Cellular responses to stress (R-HSA-2262752)”, “hemopoiesis (GO:0030097)”, “response to nutrient levels (GO:0031667)”, “response to mechanical stimulus (GO: 0009612)” and “mitochondrion organization (GO:0007005)”. From the up-regulated pathways in organic Ranger Classic samples, one GO term was specific and related to “organophosphate biosynthetic process (GO:0090407)”.

### 3.5 Overall analysis of the impact of farming system on the Pectoralis major muscle

**Fig. 7:**
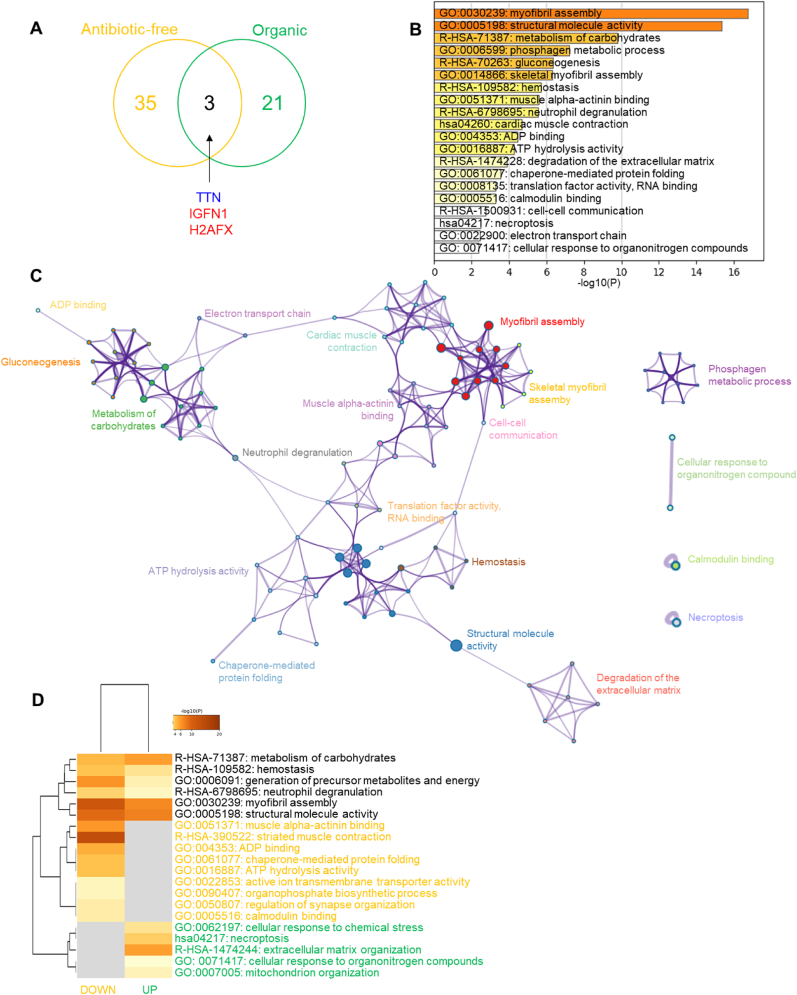
Statistical and bioinformatics analyses on the differentially expressed proteins between Antibiotic-free (DEPs = 38) and Organic (DEPs = 24) chicken *post-mortem* muscle proteome within the strains. **A)** Venn diagram highlights the number of common proteins identified. In blue down-regulated and in in red up-regulated proteins in Ranger Classic chickens respect to Ross 308. **B-D)** Bioinformatic enrichment analyses (Gene Ontology, KEGG, Reactome) on the 59 DEPs. **B)** Top enriched terms. **C)** Network layout based on the pathways of the 59 DEPs. Each term is represented by a circle node, where its size is proportional to the number of input genes fall under that term, and its color represent its cluster identity. Terms with a similarity score >0.3 are linked by an edge (the thickness of the edge represents the similarity score). **D)** Hierarchical Heatmap clustering comparing the UP (n = 24) and DOWN (n = 38) DEPs in terms of the significant process and pathways among the top Gene Ontology terms and colored according to P-values: terms with a P-value <0.01, a minimum count of 3, and an enrichment factor >1.5. Colors from grey to brown indicate p-values from high to low; and grey cells indicate the lack of significant enrichment. The terms in yellow color are specific to down-regulated proteins, those in green are for up-regulated proteins in organic chickens and those in black are significant and common to both protein lists. (For interpretation of the references to color in this figure legend, the reader is referred to the Web version of this article.)

The overall comparison of chicken meat reared following Antibiotic-free *versus* Organic farming systems revealed 59 DEPs from which 3 were in common (Fig. 7a), these being titin (TTN), immunoglobulin-like and fibronectin type III domain-containing protein 1 (IGFN1) and the histone H2AFX (H2AFX). TTN was up-regulated in Ranger Classic when compared to Ross 308 whatever the farming system, while IGFN1 and H2AFX were down-regulated in the same conditions.

The bioinformatics enrichment analyses using the list of 59 proteins by means of GO, KEGG and Reactome databases are given in Fig. 7b. Twenty GO terms were significantly enriched, which were mainly dominated by “myofibril assembly (GO:0030239)” and “structural molecule activity (GO:0005198)” followed by “metabolism of carbohydrates (R-HSA-71387)”, “phosphagen metabolic process (GO:0006599)” and skeletal myofibril assembly (GO:0014866). These enriched cluster terms allowed to construct an interconnected process network of these underlying pathways (Fig. 7c). It confirmed the dominance of a sub-network related to structural molecules activity, muscle structure and contraction and another one related to metabolic processes and ATP hydrolysis activity, but all were interacting with several other pathways. The heatmap comparing the up- and down-regulated proteins in terms of enriched terms is given in Fig. 7d. The common and specific biological pathways to the two protein lists are evidenced. Six enriched terms were found to be common being “metabolism of carbohydrates (R-HSA-71387)”, “hemostasis (R-HSA-109582)”, “generation of precursor metabolites and energy (GO:0006091)”, “neutrophil degranulation (R-HSA-6798695)”, “myofibril assembly (GO:0030239)” and “structural molecule activity (GO:0005198)”. Nine pathways were specific and down-regulated in organic chicken samples (up-regulated in Antibiotic-free samples): “muscle alpha-actinin binding (GO:0051371)”, “striated muscle contraction (R-HSA-390522)”, “ADP binding (GO:004353)”, “chaperone-mediated protein folding (GO:0061077)”, “ATP hydrolysis activity (GO:0016887)”, “active ion transmembrane transporter activity (GO:0022853)”, “organophosphate biosynthetic process (GO:0090407)”, “regulation of synapse organization (GO:0050807)” and “calmodulin binding (GO:0005516)”. However, five pathways were specific and up-regulated in organic chicken samples (down-regulated in Antibiotic-free samples), these being “cellular response to chemical stress (GO:0062197)”, “necroptosis (hsa04217)”, “extracellular matrix organization (R-HSA-1474244)”, “cellular response to organonitrogen compounds (GO: 0071417)” and “mitochondrion organization (GO:0007005)”

### 3.6 Overall analysis of the impact of chicken strain on the Pectoralis major muscle

**Fig. 8:**
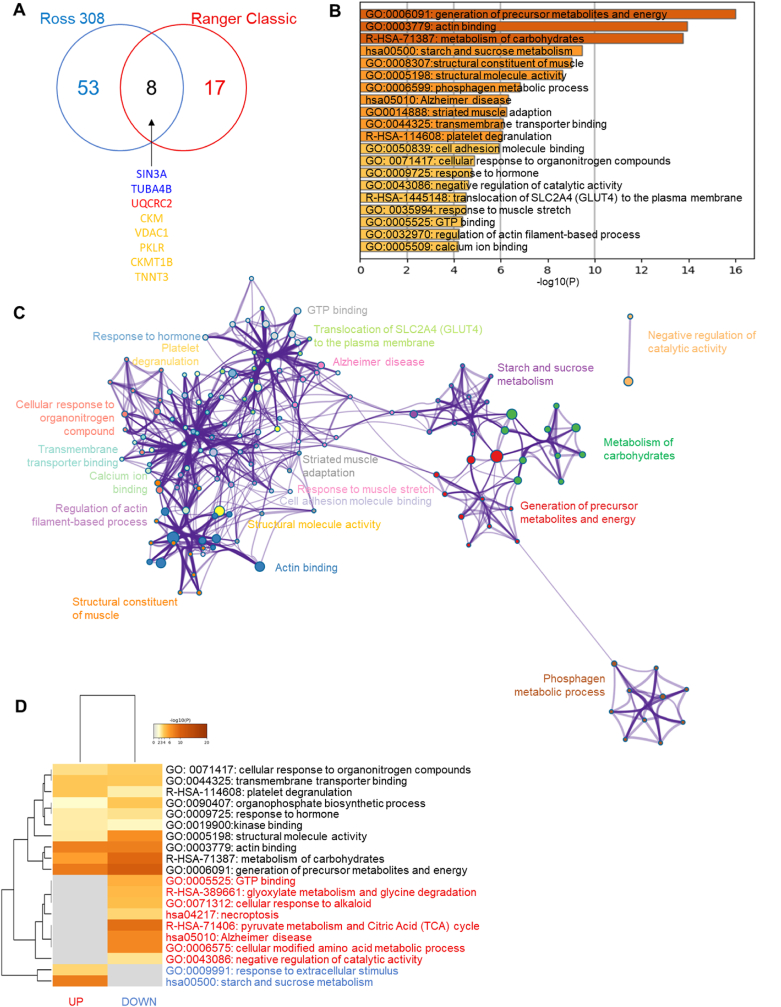
Statistical and bioinformatics analyses on the differentially expressed proteins between Ross 308 (DEPs = 61) and Ranger Classic (DEPs = 25) chicken *post-mortem* muscle proteome within the farming methods. **A)** Venn diagram highlights the number of common proteins identified. Gene names highlighted in blue are down-regulated and in red are up-regulated in organic samples respect to antibiotic-free, while proteins in yellow follow different directions. **B-D)** Bioinformatic enrichment analyses (Gene Ontology, KEGG, Reactome) on the 78 DEPs. **B)** Top enriched terms. **C)** Network layout based on the pathways of the 78 DEPs. Each term is represented by a circle node, where its size is proportional to the number of input genes fall under that term, and its color represent its cluster identity. Terms with a similarity score >0.3 are linked by an edge (the thickness of the edge represents the similarity score). **D)** Hierarchical Heatmap clustering comparing the UP (n = 34) and DOWN (n = 49) DEPs in terms of the significant process and pathways among the top Gene Ontology terms and colored according to P-values: terms with a P-value <0.01, a minimum count of 3, and an enrichment factor >1.5. Colors from grey to brown indicate p-values from high to low; and grey cells indicate the lack of significant enrichment. The terms in blue color are specific to down-regulated proteins, those in red are for up-regulated proteins in Ranger Classic samples and those in black are significant and common to both protein lists. (For interpretation of the references to color in this figure legend, the reader is referred to the Web version of this article.)

The comparison of chicken meat from two different chicken strains (Ranger Classic *versus* Ross 308), revealed 61 DEPs from which 8 were in common (Fig. 8a), these being paired amphipathic helix protein Sin3a (SIN3A), putative tubulin-like protein alpha-4B (TUBA4B), cytochrome *b*-*c*1 complex subunit 2 (UQCRC2), creatine kinase M-type (CKM), voltage-dependent anion-selective channel protein 1 (VDAC1), pyruvate kinase (PKLR), creatine kinase U-type (CKMT1B) and troponin T (TNNT3). SIN3A and TUBA4B were up-regulated and UQCRC2 was down-regulated in organic samples, while the other proteins were in both directions (up and down) depending on the farming system.

The bioinformatics enrichment analyses on the 61 DEPs through Gene Ontology (GO), KEGG and Reactome databases are given in Fig. 8b. It resulted that 20 cluster terms were significantly enriched mainly dominated by “generation of precursor metabolites and energy (GO:0006091)”, “actin binding (GO:0003779)” and metabolism of carbohydrates (R-HSA-71387) followed by “starch and sucrose metabolism (hsa00500)”, structural constituent of muscle (GO:0008307)”, “structural molecule activity (GO:0005198)” and “phosphagen metabolic process (GO:0006599)” as top 7 terms. These enriched cluster terms allowed to construct a process network of the pathways (Fig. 8c). It confirmed the dominance of a sub-network related to metabolic processes, structural molecules, and actin binding activities.

The comparison by means of a heatmap of the up- and down-regulated proteins in terms of enriched terms is given in Fig. 8d. The analysis revealed ten common enriched terms: “cellular response to organonitrogen compounds (GO: 0071417)”, “transmembrane transported binding (GO:0044325)”, “platelet degranulation (R-HAS-114608)”, “organophosphate biosynthetic process (GO:0090407)”, “response to hormone (GO:0009725)”, “kinase binding (GO:0019900)”, “structural molecule activity (GO:0005198)”, “actin binding (GO:0003779)”, “metabolism of carbohydrates (R-HSA-71387)” and “generation of precursor metabolites and energy (GO:0006091)”. Eight GO terms were specific and up-regulated in Ranger Classic meat being “GTP binding (GO: 0005525)”, “glyoxylate metabolism and glycine degradation (R-HSA-389661)”, “cellular response to alkaloid (GO:0071312)”, “necroptosis (hsa04217)”, “pyruvate metabolism Citric Acid (TCA) cycle (R-HSA-71406)”, “Alzheimer disease (hsa05010)”, “cellular modified amino acid metabolic process (GO:0006575)” and “negative regulation of catalytic activity (GO:0043086)”. However, two GO terms were specific and down-regulated in Ranger Classic being: “response to extracellular stimulus (GO:0009991)” and “starch and sucrose metabolism (hsa00500)”.

## 4 Discussion

In this trial, the lofty goal of candidate protein biomarkers identification associated with differences in chicken farming systems and/or strains was achieved using a shotgun proteomic approach. The applied shotgun in the frame of SWATH-MS proteomics technique has been confirmed as a powerful tool to study the early post-mortem muscle proteome taken from the chicken samples. The findings presented above, revealed for the first-time new pathways and novel insights to understand differences between the two farming systems within the two strains we considered in this trial.

The comparison of the muscle proteome of ARA and ARO, revealed 38 potential biomarkers. The robustness of the obtained list of proteins as putative biomarkers was confirmed by the statistical analysis we applied. The dominance of sub-network related to muscle structure, contraction, and associated pathways, as well as another one related to ATP and energy metabolic processes emerged from the bioinformatics analyses. In fact, the comparison of the muscle proteome of ORA and ORO, revealed a smaller number of changing proteins. Unlike the previous comparison between the same strains reared under an antibiotic-free production system, in this case there was no clear separation between the two groups, both in the PCA plot and in the hierarchical clustering analysis, with only a few individuals being distinguishable from each other. This was further confirmed by the heatmap analysis, which showed a significant overlap between the two groups. Bioinformatics enrichment analyses revealed 11 significantly enriched cluster terms, with the dominant terms being "sarcomere organization" and "structural molecule activity," followed by "metabolism of carbohydrates" and "cellular response to stress". Skeletal muscle proteins were found to play a crucial role in the characterization of Italian chicken breeds being MYL an important marker (Zanetti et al. 2011). A similar result was obtained in an earlier study that compared the muscle proteomes of commercial broilers and Leghorn chickens (Zapata et al. 2012). The results indicated a shift in the energy metabolism in muscle growth, more specifically in commercial broilers, being a fast-growing breed. Other studies evidenced that the differences can be ascribed to the intrinsic response of the genotypes used and to the kinetic behavior, which is prerequisite for the adaptation of the animals (Mattioli et al. 2021). A former study by De Liu and co-workers reported that the muscle structure protein expressions, especially myosins, can contribute to meat quality traits and breed specific meat flavor (De Liu et al. 2012). This supports part of the findings we observed in this study. The enriched cluster terms allowed the construction of a process network of pathways, which further confirmed the dominance of a sub-network of sarcomere organization and structural molecule activity pathways.

The comparison of the up- and down-regulated proteins in terms of enriched terms revealed that one enriched term was common to both protein lists, which was related to "cellular response to stress". Cellular response to stress is a pivotal pathway in post-mortem muscle (Lamri *et al.* 2023a), which is further identified in poultry research to be key, especially within the frame of mitochondria as major sites of oxidative processes related to both fat oxidation and energy production (Lauridsen 2019). This pathway was reported using proteomics to be significant in meat color and tenderness determination of several meat quality traits (tenderness, color, water-holding-capacity, …) and across diverse species (López-Pedrouso et al. 2020; Gagaoua *et al.* 2021a; Purslow et al. 2021; Gagaoua & Picard 2022). On another hand, it is well admitted that the extent and, hence, the negative consequences of the oxidative stress in the living birds and their muscles can be modulated by livestock handling and by dietary means (Estévez 2015). Moreover, oxidative stress was described to impair meat quality of broiler by damaging mitochondrial function, affecting calcium homeostasis and leading to apoptosis (Chen et al. 2022).

The comparison of the muscle proteome of Ross 308 chicken meat reared under Antibiotic-free and Organic allowed achieving a clear separation. The top enriched cluster terms were related to pathways involved in energy metabolism and glycolytic pathways likely “carbonic metabolism”, “pyruvate metabolic process”, “pyruvate metabolism and Citric Acid (TCA) cycle” in interplay with pathways from the muscle structure such as “muscle system process”, “actin binding”, and “structural constituent of cytoskeleton” among others. A previous study assessed that glycolysis-related pathways showed a positive correlation with the meat texture as they were significantly up-regulated in chicken meat samples that reported a lower shear force (Teltathum & Mekchay 2009). Similarly, Mekchay *et al.* used the same approach to compare the Thai local chicken and commercial broiler, confirming the results (Mekchay et al. 2010). Other studies have confirmed that glycolytic proteins have a high impact on meat tenderness from different animal origin (Gagaoua et al. 2020; Picard & Gagaoua 2020; Gagaoua *et al.* 2021a). This indicates that glycolytic proteins (glycolytic metabolism) can be used as a key molecular signature for chicken meat quality monitoring and authenticity for the origin purposes, for instance, for the type of farming system. Previous research on cattle further evidenced the potential of protein biomarkers evaluated by Dot-Blot, a simple and convenient method for detection of proteins in crude lysates or extracts without the need for separation by SDS-PAGE, to discriminate among rearing practices (Gagaoua et al., 2017).

Finally, the comparison of the muscle proteome of Ranger Classic chicken meat reared under antibiotic-free (ARA) and organic (ORA) farming systems revealed 25 putative biomarkers with an acceptable level of separation using both PCA and/or clustering analyses. The SIN3A transcriptional regulatory protein and myosin heavy chains (MYH1A and MYH1C) resulted to be the proteins with the highest fold change. Myosin heavy chain were reported to be negatively correlated with Warner-Bratzler shear force of broiler breast meat (Desai et al. 2016). In our comparison, the “generation of precursor metabolites and energy” and “sarcomere organization” were the most changing molecular signatures. These results suggest that the organic production system may affect the energy metabolic processes and muscle structure of Ranger Classic chickens. The process network of the pathways further confirmed the dominance of a sub-network related to muscle structure and associated pathways and another one related to energy metabolic processes. Taken together, the results suggest that organic production system may significantly affect the muscle proteome of Ranger Classic chickens, particularly in terms of energy metabolic processes, muscle structure, and actin cytoskeleton organization. However, further studies are needed to fully understand the molecular mechanisms underlying these differences and their potential impact on meat quality and nutritional value of the produced meat. The proposed lists of protein biomarkers belong to specific molecular signatures that are worthy of validation under other sampling conditions and experimental design before the setup of a targeted proteomics for the evaluation for the proposed biomarkers.

The organic farming system led to differential expression of proteins involved in various metabolic pathways such as carbon and pyruvate metabolism, as well as pathways related to muscle system and structure. Moreover, the comparison of Ross 308 and Ranger Classic strains evidenced significant differences in the muscle proteome, these are partly related to their genetic background but, according to our findings, are also influenced by rearing conditions. Overall, these findings suggest that different rearing systems can have a significant impact on the microbiota, metabolites, and proteome of chicken meat, which may have implications for the nutritional and sensory properties of the meat, as well as for food safety. This study may have important implications for the poultry industry, as it highlights the importance of genetic selection and management practices in impacting the muscle proteome, and possibility in improving the quality of chicken meat. According to a recent study, slow-growing chicken genotypes that are relatively small at 81 days of age (the body weight that consumers expect) and have desirable feed conversion ratio values and moderate unit costs are the best compromise in organic farming from an economic standpoint (Obremski et al. 2023). On the other hand, achieving specific biomarkers which can be used to detect food frauds could help industries in justifying the higher price of organic meat and guarantee to consumers the authenticity of the product they are buying. Undoubtedly, the knowledge on this field is still at the beginning, but by carrying out more research in this sense, it could lead, in the next years, to the production of simple analytical tools accessible to companies and/or distributors to ascertain the authenticity.

Further investigations using other in-depth omics approaches, such metabolomics, would help achieve in the frame of a multi-omics approach a better understanding of the underlying mechanisms partly revealed in this trial as well as accurately refining the candidate biomarkers. In fact, metabolomics approach involves qualitative and quantitative measurement of metabolites through untargeted fingerprinting and targeted profiling of the muscle/meat matrix (Muroya et al. 2020; Shi et al. 2022; Zhang et al. 2023). We suppose that this approach can give information about metabolic responses to the changes in genetic and environmental conditions and it could accurately characterize the physiological state of the organisms as previously evidenced in poultry research (Tan et al. 2023). In this scenario, a metabolomics approach would be interesting to undertake to better understand the mechanisms of specific pathways related to chicken strains and farming systems. The integration of metabolomics with the proteomics data obtained in this research will increase the knowledge about chicken meat determination at the molecular level and will help to have a more comprehensive view to connect the genotype and the external environment (farming practices) on the final quality meat product (including meat quality traits, meat flavor, shelf-life, authenticity). This should further improve the ideal decision tools' ability to provide accurate prediction.

## 5 Conclusions

The present study represents the first application of a proteomics approach using SWATH-MS in studying the impact of organic farming on early post-mortem chicken muscle. The proteomes of four groups of *Pectoralis major* muscles of two chicken strains from both organic and non-organic farming were investigated. The starting hypotheses and aims were achieved confirming the crucial impact, not only of chicken strain but also of farming system on chicken muscle proteomes and probably of the differences in breast meat qulaity traits. In particular, the data showed high similarities in proteome revealing that the organic farming can influence the muscle proteome more than the conventional farming system, i.e., antibiotic-free production system. Achieving knowledge about the impact of the farming and production methods is meant to be an initial step towards the application of these techniques in routine production of chicken meat. Moreover, this study reports a first screening for putative biomarkers identification which need to be further evaluated using two methods. First, chemometrics and statistical methods would be very useful to evaluate the accuracy of the proteins to discirminate between the groups using for instance Partial Least Squares Discriminant Analysis (PLS-DA) or Artificial Neural Networks (ANN), which is a deep learning algorithm inspired by biological neural networks. Second, the candidate biomarkers warrant to be confirmed by targeted proteomics approaches such as as Multiple Reaction Monitoring (MRM) and parallel reaction monitoring (PRM). The ultimate goal is the possibility that these foodomics methods, with the necessary improvements, could be adopted, in a near future, by surveillance bodies or quality control departments of chicken meat industries to combat frauds or provide more precise labels for organically produced food of animal origin.

## CRediT authorship contribution statement

**Laura Alessandroni:** Writing – original draft, Investigation, Methodology, Formal analysis, Visualization, Data curation, Conceptualization. **Gianni Sagratini:** Writing – review & editing, Validation, Supervision, Project administration, Funding acquisition, Conceptualization. **Susana B. Bravo:** Software, Methodology, Investigation. **Mohammed Gagaoua:** Writing – review & editing, Writing – original draft, Visualization, Validation, Supervision, Software, Resources, Methodology, Investigation, Formal analysis, Data curation, Funding acquisition, Conceptualization.

## Declaration of competing interest

The authors declare no conflict of interest.

## Data Availability

Data will be made available on request.

## References

[bib2] Alessandroni L., Caprioli G., Faiella F., Fiorini D., Galli R., Huang X., Marinelli G., Nzekoue F., Ricciutelli M., Scortichini S., Sagratini G. (2022). A shelf-life study for the evaluation of a new biopackaging to preserve the quality of organic chicken meat. Food Chem..

[bib4] Alessandroni L., Scortichini S., Caprioli G., Fiorini D., Huang X., Silvi S., Galli R., Sagratini G. (2023). Assessing chemical, microbiological and sensorial shelf-life markers to study chicken meat quality within divergent production systems (organic vs. conventional). Eur. Food Res. Technol..

[bib3] Alessandroni L., Sagratini G., Gagaoua M. (2024). Proteomics and bioinformatics analyses based on two-dimensional electrophoresis and LC-MS/MS for the primary characterization of protein changes in chicken breast meat from divergent farming systems: organic versus antibiotic-free. Food Chem.: Molecular Sciences.

[bib1] Aviagen Group (2018). Ranger Classic Broiler Performance Objectives.

[bib5] Babicki S., Arndt D., Marcu A., Liang Y., Grant J.R., Maciejewski A., Wishart D.S. (2016). Heatmapper: web-enabled heat mapping for all. Nucleic Acids Res..

[bib6] Bonzon-Kulichenko E., Pérez-Hernández D., Núñez E., Martínez-Acedo P., Navarro P., Trevisan-Herraz M., del Carmen Ramos M., Sierra S., Martínez-Martínez S., Ruiz-Meana M. (2011). A robust method for quantitative high-throughput analysis of proteomes by 18O labeling. Mol. Cell. Proteomics.

[bib7] Bouley J., Chambon C., Picard B. (2004). Mapping of bovine skeletal muscle proteins using two-dimensional gel electrophoresis and mass spectrometry. Proteomics.

[bib8] Bradford M.M. (1976). A rapid and sensitive method for the quantitation of microgram quantities of protein utilizing the principle of protein-dye binding. Anal. Biochem..

[bib9] Chen Z., Xing T., Li J., Zhang L., Jiang Y., Gao F. (2022). Oxidative stress impairs the meat quality of broiler by damaging mitochondrial function, affecting calcium metabolism and leading to ferroptosis. Anim Biosci.

[bib11] De Liu X., Jayasena D.D., Jung Y., Jung S., Kang B.S., Heo K.N., Lee J.H., Jo C. (2012). Differential proteome analysis of breast and thigh muscles between Korean native chickens and commercial broilers. Asian-Australas. J. Anim. Sci..

[bib12] della Malva A., Gagaoua M., Santillo A., De Palo P., Sevi A., Albenzio M. (2022). First insights about the underlying mechanisms of Martina Franca donkey meat tenderization during aging: a proteomic approach. Meat Sci..

[bib13] della Malva A., Maggiolino A., De Palo P., Albenzio M., Lorenzo J.M., Sevi A., Marino R. (2022). Proteomic analysis to understand the relationship between the sarcoplasmic protein patterns and meat organoleptic characteristics in different horse muscles during aging. Meat Sci..

[bib14] Demichev V., Messner C.B., Vernardis S.I., Lilley K.S., Ralser M. (2020). DIA-NN: neural networks and interference correction enable deep proteome coverage in high throughput. Nat. Methods.

[bib15] Desai M.A., Jackson V., Zhai W., Suman S.P., Nair M.N., Beach C.M., Schilling M.W. (2016). Proteome basis of pale, soft, and exudative-like (PSE-like) broiler breast (Pectoralis major) meat. Poultry Sci..

[bib16] Estévez M. (2015). Oxidative damage to poultry: from farm to fork. Poultry Sci..

[bib10] European Commission. (1235/2008) Laying Down Detailed Rules for Implementation of Council Regulation (EC) No 834/2007 as Regards the Arrangements for Imports of Organic Products from Third Countries.

[bib17] Feknous I., Saada D.A., Boulahlib C.Y., Alessandroni L., Souidi S.W., Chabane O.A., Gagaoua M. (2023). Poultry meat quality preservation by plant extracts: an overview. Sci. J. Meat Technol..

[bib61] Gagaoua M., Monteils V., Couvreur S., Picard B. (2017). Identification of biomarkers associated with the rearing practices, carcass characteristics, and beef quality: An integrative approach. J. Agric. Food Chem..

[bib18] Gagaoua M., Hughes J., Terlouw E.C., Warner R.D., Purslow P.P., Lorenzo J.M., Picard B. (2020). Proteomic biomarkers of beef colour. Trends Food Sci. Technol..

[bib19] Gagaoua M., Picard B., Purslow P. (2022). New Aspects of Meat Quality.

[bib20] Gagaoua M., Schilling W.M., Zhang X., Suman S.P. (2024). Encyclopedia of Meat Sciences.

[bib21] Gagaoua M., Suman S.P., Purslow P.P., Lebret B. (2023). The color of fresh pork: consumers expectations, underlying farm-to-fork factors, myoglobin chemistry and contribution of proteomics to decipher the biochemical mechanisms. Meat Sci..

[bib22] Gagaoua M., Terlouw E.C., Mullen A.M., Franco D., Warner R.D., Lorenzo J.M., Purslow P.P., Gerrard D., Hopkins D.L., Troy D. (2021). Molecular signatures of beef tenderness: underlying mechanisms based on integromics of protein biomarkers from multi-platform proteomics studies. Meat Sci..

[bib23] Gagaoua M., Troy D., Mullen A.M. (2021). The extent and rate of the appearance of the major 110 and 30 kDa proteolytic fragments during post-mortem aging of beef depend on the glycolysing rate of the muscle and aging time: an LC-MS/MS approach to decipher their proteome and associated pathways. J. Agric. Food Chem..

[bib24] Gagaoua M., Warner R.D., Purslow P., Ramanathan R., Mullen A.M., López-Pedrouso M., Franco D., Lorenzo J.M., Tomasevic I., Picard B., Troy D., Terlouw E.M.C. (2021). Dark-cutting beef: a brief review and an integromics meta-analysis at the proteome level to decipher the underlying pathways. Meat Sci..

[bib25] Karlsson L. (2016).

[bib26] Kaygisiz F., Bolat B.A., Bulut D. (2019). Determining factors affecting consumer's decision to purchase organic chicken meat. Brazilian J. Poultry Sci..

[bib27] Lamri M., della Malva A., Djenane D., Albenzio M., Gagaoua M. (2023). First insights into the dynamic protein changes in goat Semitendinosus muscle during the post-mortem period using high-throughput proteomics. Meat Sci..

[bib28] Lamri M., della Malva A., Djenane D., López-Pedrouso M., Franco D., Albenzio M., Lorenzo J.M., Gagaoua M. (2023). Towards the discovery of goat meat quality biomarkers using label-free proteomics. J. Proteonomics.

[bib29] Lauridsen C. (2019). From oxidative stress to inflammation: redox balance and immune system. Poultry Sci..

[bib30] López-Pedrouso M., Lorenzo J.M., Gagaoua M., Franco D. (2020). Application of proteomic technologies to assess the quality of raw pork and pork products: an overview from farm-to-fork. Biology.

[bib31] Luo L., Zhang Q., Kong X., Huang H., Ke C. (2017). Differential effects of bisphenol A toxicity on oyster (Crassostrea angulata) gonads as revealed by label-free quantitative proteomics. Chemosphere.

[bib32] Mattioli S., Cartoni Mancinelli A., Menchetti L., Dal Bosco A., Madeo L., Guarino Amato M., Moscati L., Cotozzolo E., Ciarelli C., Angelucci E., Castellini C. (2021). How the kinetic behavior of organic chickens affects productive performance and blood and meat oxidative status: a study of six poultry genotypes. Poultry Sci..

[bib33] Mekchay S., Teltathum T., Nakasathien S., Pongpaichan P. (2010). Proteomic analysis of tenderness trait in Thai native and commercial broiler chicken muscles. J. Poultry Sci..

[bib34] Meyer J.G., Schilling B. (2017). Clinical applications of quantitative proteomics using targeted and untargeted data-independent acquisition techniques. Expet Rev. Proteonomics.

[bib35] Muroya S., Ueda S., Komatsu T., Miyakawa T., Ertbjerg P. (2020). MEATabolomics: muscle and meat metabolomics in domestic animals. Metabolites.

[bib36] Nguyen H.V., Nguyen N., Nguyen B.K., Lobo A., Vu P.A. (2019). Organic food purchases in an emerging market: the influence of consumers' personal factors and green marketing practices of food stores. Int. J. Environ. Res. Publ. Health.

[bib37] Obremski K., Tyburski J., Wojtacha P., Sosnówka-Czajka E., Skomorucha I., Pomianowski J., Parowicz P. (2023). Assessment of the economic profitability of fattening selected chicken genotypes in an organic farm. Agriculture.

[bib38] Ortea I., González-Fernández M.a.J., Ramos-Bueno R.P., Guil-Guerrero J.L. (2018). Proteomics study reveals that docosahexaenoic and arachidonic acids exert different in vitro anticancer activities in colorectal cancer cells. J. Agric. Food Chem..

[bib39] Picard B., Gagaoua M. (2020). Meta-proteomics for the discovery of protein biomarkers of beef tenderness: an overview of integrated studies. Food Res. Int..

[bib41] Purslow P.P., Gagaoua M., Warner R.D. (2021). Insights on meat quality from combining traditional studies and proteomics. Meat Sci..

[bib42] Rocchi L., Cartoni Mancinelli A., Paolotti L., Mattioli S., Boggia A., Papi F., Castellini C. (2021). Sustainability of rearing system using multicriteria analysis: application in commercial poultry production. Animals.

[bib43] Roy A., Ghosh A., Vashisht D. (2023). The consumer perception and purchasing attitude towards organic food: a critical review. Nutr. Food Sci..

[bib44] Schwartzkopf-Genswein K., Faucitano L., Dadgar S., Shand P., González L., Crowe T. (2012). Road transport of cattle, swine and poultry in North America and its impact on animal welfare, carcass and meat quality: a review. Meat Sci..

[bib45] Shevchenko A., Jensen O.N., Podtelejnikov A.V., Sagliocco F., Wilm M., Vorm O., Mortensen P., Shevchenko A., Boucherie H., Mann M. (1996). Linking genome and proteome by mass spectrometry: large-scale identification of yeast proteins from two dimensional gels. Proc. Natl. Acad. Sci. USA.

[bib46] Shi K., Zhao Q., Shao M., Duan Y., Li D., Lu Y., Tang Y., Feng C. (2022). Untargeted metabolomics reveals the effect of selective breeding on the quality of chicken meat. Metabolites.

[bib47] Shilov I.V., Seymour S.L., Patel A.A., Loboda A., Tang W.H., Keating S.P., Hunter C.L., Nuwaysir L.M., Schaeffer D.A. (2007). The Paragon Algorithm, a next generation search engine that uses sequence temperature values and feature probabilities to identify peptides from tandem mass spectra. Mol. Cell. Proteomics.

[bib48] Sirri F., Castellini C., Bianchi M., Petracci M., Meluzzi A., Franchini A. (2011). Effect of fast-, medium- and slow-growing strains on meat quality of chickens reared under the organic farming method. Animal.

[bib49] Tallentire C.W., Leinonen I., Kyriazakis I. (2016). Breeding for efficiency in the broiler chicken: a review. Agron. Sustain. Dev..

[bib50] Tan C., Selamat J., Jambari N.N., Sukor R., Murugesu S., Muhamad A., Khatib A. (2023). 1H nuclear magnetic resonance‐based metabolomics study of serum and pectoralis major for different commercial chicken breeds. Food Sci. Nutr..

[bib51] Tan H.T., Chung M.C.M. (2018). Label‐free quantitative phosphoproteomics reveals regulation of vasodilator‐stimulated phosphoprotein upon stathmin‐1 silencing in a pair of isogenic colorectal cancer cell lines. Proteomics.

[bib52] Teltathum T., Mekchay S. (2009). Proteome changes in Thai indigenous chicken muscle during growth period. Int. J. Biol. Sci..

[bib53] Terlouw C., Picard B., Deiss V., Berri C., Hocquette J.-F., Lebret B., Lefèvre F., Hamill R., Gagaoua M. (2021). Understanding the determination of meat quality using biochemical characteristics of the muscle: stress at slaughter and other missing keys. Foods.

[bib54] Xing T., Gao F., Tume R.K., Zhou G., Xu X. (2019). Stress effects on meat quality: a mechanistic perspective. Compr. Rev. Food Sci. Food Saf..

[bib55] Xing T., Zhao Z., Zhao X., Zhuang S., Xu X. (2020). Phosphoproteome analysis of sarcoplasmic and myofibrillar proteins in stress-induced dysfunctional broiler pectoralis major muscle. Food Chem..

[bib56] Zaboli G., Huang X., Feng X., Ahn D.U. (2019). How can heat stress affect chicken meat quality?–a review. Poultry Sci..

[bib57] Zanetti E., Molette C., Chambon C., Pinguet J., Rémignon H., Cassandro M. (2011). Using 2‐DE for the differentiation of local chicken breeds. Proteomics.

[bib58] Zapata I., Reddish J., Miller M., Lilburn M., Wick M. (2012). Comparative proteomic characterization of the sarcoplasmic proteins in the pectoralis major and supracoracoideus breast muscles in 2 chicken genotypes. Poultry Sci..

[bib59] Zhang X., Smith S.W., Zaldivar L.R., Lesak D.J., Schilling M.W. (2023). Study of emerging chicken meat quality defects using OMICs: what do we know?. J. Proteonomics.

[bib60] Zhu Y., Gagaoua M., Mullen A.M., Viala D., Rai D.K., Kelly A.L., Sheehan D., Hamill R.M. (2021). Shotgun proteomics for the preliminary identification of biomarkers of beef sensory tenderness, juiciness and chewiness from plasma and muscle of young Limousin-sired bulls. Meat Sci..

